# Soybean products and reduction of breast cancer risk: a case–control study in Japan

**DOI:** 10.1038/sj.bjc.6602659

**Published:** 2005-06-07

**Authors:** K Hirose, N Imaeda, Y Tokudome, C Goto, K Wakai, K Matsuo, H Ito, T Toyama, H Iwata, S Tokudome, K Tajima

**Affiliations:** 1 Division of Epidemiology and Prevention, Aichi Cancer Center Research Institute, 1-1 Kanokoden Chikusa-ku, Nagoya 464-8681, Japan; 2 Nagoya Women’s University, Mizuho-ku, Nagoya 467-8610, Japan; 3 Nagoya Bunri University, Inazawa 492-8520, Japan; 4 Department of Breast Surgery, Aichi Cancer Center Hospital, 1-1 Kanokoden, Chikusa-ku, Nagoya 464-8681, Japan; 5 Department of Health Promotion and Preventive Medicine, Nagoya City University Graduate School of Medical Science, Mizuho-ku, Nagoya 467-8601, Japan

**Keywords:** soybean products, isoflavones, menopausal status

## Abstract

Components of the Japanese diet, which might contribute to the relatively low breast cancer incidence rates in Japan, have not been clarified in detail. Since soybean products are widely consumed in Japan, a case–control study taking account of the menopausal status was conducted using data from the hospital-based epidemiologic research program at Aichi Cancer Center (HERPACC). In total, 167 breast cancer cases were included and 854 women confirmed as free of cancer were recruited as the control group. Odds ratios (OR) and 95% confidence intervals (95% CI) were determined by multiple logistic regression analysis. There were reductions in risk of breast cancer associated with high intake of soybean products among premenopausal women. Compared with women in the lowest tertile, the adjusted ORs for top tertile intake of *tofu* (soybean curd) was 0.49 (95% CI, 0.25–0.95). A significant decrease in premenopausal breast cancer risk was also observed for increasing consumption of isoflavones (OR=0.44; 95% CI, 0.22–0.89 for highest *vs* lowest tertile; *P* for trend=0.02). The present study found a statistically inverse association between *tofu* or isoflavone intake and risk of breast cancer in Japanese premenopausal women, while no statistically significant association was evident with the risk among postmenopausal women.

Despite a marked increase in recent years, the incidence rates for female breast cancer in most Asian countries are much lower than those in the Western world (Parkin *et al*, 1997). Much of the international variation is due to differences in established reproductive risk factors, such as age at menarche, parity and age at first birth, but dietary habits might also contribute. Dietary studies of breast cancer have typically focused on the hypothesis that there is a positive link with intake of fat. While ecological studies have suggested associations in terms of incidence and mortality, leading prospective studies of breast cancer, including the Nurse's Health Study (Stampfer *et al*, 1987) and studies of large cohorts from New York (Toniolo *et al*, 1994) and Norway (Vatten *et al*, 1990) have shown no relation (Willett, 1997). Recently, there has been more interest in other dietary factors, such as soybean products, which may protect against breast cancer and provide an explanation for some of the international differences in incidence rates (Adlercreutz, 1990; Messina and Barnes, 1991).

Soybeans provide a unique concentrated source of isoflavones and soybeans or isoflavones have been shown to exert anticarcinogenic effects on hormone-related cancers in a large number of experimental studies. Despite the growing interest in the protective effects, there are relatively few epidemiological data available since soybean products are consumed mainly by Asian populations. In Japan, intake is in various forms, including *tofu* (soybean curd), *okara* (*tofu* lees), *moyashi* (soybean sprouts), *tonyu* (soymilk), *yuba* (soy milk skin), *kinako* (soy flour), *miso* (fermented soybean paste), *atsuage* (deep fried *tofu*), *aburage* (thinly sliced deep fried *tofu*), *natto* (fermented soybeans), *koyadofu* (freeze dried *tofu*) and *shoyu* (soy sauce), so that the diet is likely to be much richer in isoflavones than in the Western world, where the diet usually does not include soybeans. We here evaluated the association between risk of breast cancer and consumption of soybean products and isoflavones using data from the hospital-based epidemiologic research program at Aichi Cancer Center (HERPACC).

## MATERIALS AND METHODS

### Data collection

Details of the study design and subject characteristics have been described elsewhere (Yoo *et al*, 1992; Hirose *et al*, 1995, 1999, 2001). In brief, we have conducted the HERPACC study since 1988, whereby questionnaire survey is completed by first-visit outpatients to the Aichi Cancer Center Hospital (ACCH) (Tajima *et al*, 2000). All questionnaires are then collected after checking for incomplete responses by a trained interviewer and the data are loaded into the computer system of the Aichi Cancer Center Research Institute. The data collected are linked with the hospital-based cancer registry files. This study was approved by the Institutional Review Board, and all participants provided written, informed consent.

The questionnaire included questions on occupation, medical history, height, weight, weight at around 20 years of age, family history (parents and siblings), smoking and drinking habits, sleeping habits, physical exercise and reproductive history. The details were taken prior to assessment of symptoms and all information was collected before clinical diagnoses were made.

### Cases and controls

The present analysis was restricted to women aged 30 and over who visited hospitals between January 2001 and March 2002. As eligible cases, 79 premenopausal and 88 postmenopausal women diagnosed as having breast cancer by histological examination within 6 months of the first-visit were recruited. A methodological study applying the same HERPACC data set earlier showed that the OR based on a large number of controls gives more power and steadier estimates than the use of matched controls (Hamajima *et al*, 1994); therefore, we used all noncancer individuals as candidates for controls in this study. As the controls, 854 female first-visit outpatients who had never been diagnosed as having cancer were recruited. Table 1 summarises details for the 167 cases and 854 controls by age group and selected characteristics.

### Exposure data

The interview included a validated, semiquantitative food-frequency questionnaire (FFQ) (Tokudome *et al*, 1998, 2001; Imaeda *et al*, 2002). Dietary intake was ascertained using a detailed quantitative FFQ, including 119 food items/recipes, and the following food groups were included: (a) meat and meat products (poultry, ground meat, pork meat, beef, ham, sausage, bacon, liver); (b) fish and fish products (salmon, eel, pale blue fleshed fish, red fleshed fish, white fleshed fish, squid, octopus, shrimp, crab, dried fish larvae, small bony fish, tuna canned in oil, cod roe, oyster, shellfish, dried squid, fried fish paste, fish paste sausage); (c) green-yellow vegetables (green leafy vegetables, pumpkin, carrot, broccoli, green pepper, green soybean, green beans, tomato); (d) other vegetables (cabbage, Japanese radish, burdock, bamboo shoots, cucumber, eggplant, lettuce, bean sprout, onion, Chinese cabbage, Japanese radish); (e) fruits (oranges, mandarin oranges, persimmon, banana, apple, strawberries, kiwi, peach, grapes, watermelon, melon, Japanese pears); (f) dairy products (low fat milk, medium fat milk, high fat milk, calcium enriched milk and yoghurt, skim milk, lactic acid bacteria beverage, yoghurt, cheese, ice cream).

Furthermore, the following soybean products were included: (a) *tofu* (soybean curd); (b) *miso* (fermented soybean paste); (c) *atsuage* (deep fried *tofu*); (d) *aburage* (thinly sliced deep fried *tofu*); (e) *natto* (fermented soybeans); (f) *koyadofu* (freeze dried *tofu*). We evaluated validity of intakes based on the questionnaire against those according to 28-day (four-season consecutive 7-day) weighted diet records among 79 Japanese female dietitians. In the validation study, the Spearman’s correlation coefficients between the estimate intake of soybean products and isoflavone from the questionnaire and that from dietary records were 0.51, 0.53, respectively. For reproducibility of estimation from the questionnaire, the Spearman’s correlation coefficients for the consumption of soybean products and isoflavone intake between two questionnaires administered 1 year apart were 0.57 and 0.47, respectively.

All subjects in the present study were asked for average frequency and portion size of consumption, during the period of 1 year before onset of the present disease or before the interview. There were eight categories of possible responses, ranging from‘rarely or never’ to ‘three or more times per day’. For each food, a commonly used unit or portion size was specified and the interviewers asked the subjects using sampling models of full-scale photographs. We ascertained average amount of daily consumption of each food and nutrients by multiplying the food intake (in grams) or serving size and the nutrient content per 100 grams of food as listed in the Standard Tables of Food Composition, Version 5 and the Follow-up of Standard Tables of Food Composition (Science and Technology Agency, Japan, 1994, 2001). Isoflavone intakes were separately calculated from USDA-Iowa State University Database on the Isoflavone Content of Foods, Release 1.3-2002 (http://www.nal.usda.gov/fnic/foodcopm/Data/isoflav/isoflav.html). Isoflavone intake was calculated using consumption of six items of soybean products, green soybean, peanuts, Japanese green tea, and vegetables other than green-yellow vegetables such as cucumber, eggplant, lettuce, bean sprouts, onion, Chinese cabbage.

### Statistical analysis

Dietary intake data were analysed by individual food items, food groups and nutrients for all subjects combined and separately for premenopausal and postmenopausal women. The differences of means were examined by *t*-test and all *P*-values presented are two-sided.

Logistic regression analysis was used to obtain odds ratios (ORs) and 95% confidence intervals (95% CI) as estimates of relative risk. The *P*-value for trend corresponded to the estimate of the slope derived from the logistic model in the case that the integers, 1 to *n*, were assigned to the ordered *n* levels of each factors. The LOGISTIC procedure provided by SAS (SAS Institute, Cary, NC, USA) was utilised to perform the calculations. To compare differences and similarities of effects of Japanese diet on risk for breast cancer, subjects were stratified with reference to menopausal status. The ORs of breast cancer are calculated for the selected food groups and isoflavone intake in tertiles, with the lowest tertile as the referents. Multivariate models was adjusted for age, energy (as a continuous variable), motives for consultation (self-recommendation, family recommendation, referral from other clinics, secondary screening after primary screening, others), smoking status (never, ever, current), drinking status (never, ever, current), exercise (none, ⩽60 min/week, ⩽120 min/week, >120 min/week), family history (yes or no), age at menarche (⩽12, 13, ⩾14), parity (0,1,2, 3+), age at first full-term pregnancy (⩽23, 24–27, ⩾28), and current body mass index (BMI) (⩽20, 20–25, ⩾25). Further, inclusion in the model of age at menopause (⩽47, 48–52, ⩾53) was performed with the calculations for postmenopausal women. Energy was adjusted for the multivariate nutrient density method.

A positive family history may involve different types and numbers of relatives. Distant relatives share less genetic influence and fewer confounding environmental and/or behavioral factors than do close ones. Furthermore, information on the medical history of distant relatives is limited and less precise than that of close or first-degree relatives. In this study, the presence of either a mother or sister with breast cancer was considered as a positive family history. BMI was calculated as weight/height^2^ (kg m^−2^), according to Quetelet's formula and current BMI values were stratified into three categories. Since a BMI⩾25 is defined as obese by the Japanese Society for the Study of Obesity, the cutoff for the highest BMI group was BMI⩾25.

## RESULTS

Table 2 summarises data for daily intake of some nutrients and consumption of selected food groups among cases and controls. There were no significant differences between cases and controls in intake of energy and fat. The means of meat and meat products intakes per day was 60.7 g (standard deviation (s.d.), 34.7 g) and 67.0 g (s.d., 39.4 g), respectively, for cases and controls (*P*<0.04). Among premenopausal women, the means of soybean products were 51.7 *g* (s.d., 31.2 g) in case group and 63.5 g (s.d., 38.8 g) in control group (*P*<0.01, data not shown). Breast cancer cases reported lower total isoflavones intake per day than controls, with averages of 20.8 mg (s.d., 10.8 mg) and 25.8 mg (s.d., 14.3 mg), respectively (*P*<0.0001, data not shown) among premenopausal women. We observed no other significant differences among premenopausal women and there were no significant differences between cases and controls in average intake of food groups and nutrients among postmenopausal women.

Among premenopausal women, breast cancer risk was inversely associated with consumption of soybean products (Table 3). The ORs was 0.53 (95% CI, 0.27–1.04) for the top tertile of soybean product intake compared with the lowest tertile of intake (trend test, *P*=0.06). Among postmenopausal women, on the other hand, consumptions of soybean products, meat and meat products, fish and fish products, vegetables, fruits, dairy products were not associated with the risk of breast cancer.

The ORs for breast cancer according to type of soybean product are presented in Table 4. A statistically significant inverse relation was observed between *tofu* consumption and breast cancer in premenopausal women. ORs were 0.44 (95% CI, 0.22–0.90), 0.49 (95% CI, 0.25–0.95) for the second to the top tertiles of intake compared with the lowest tertile of intake of *tofu* (trend test, *P*=0.03). Compared with those in the lowest tertile of *atsuage* (deep fried tofu) consumption, the adjusted OR for breast cancer in top tertile was 0.70 (95% CI, 0.35–1.38) among premenopausal women. On the other hand, a significantly increased risk of breast cancer with consumption of *atsuage* was observed in postmenopausal women. The adjusted OR for top tertile of *atsuage* consumption was 2.28 (95% CI, 1.15–4.51, trend test *P*=0.02). Also, consumption of *aburage* (thinly sliced deep fried tofu) showed a similar positive association with the risk of breast cancer. There were no associations with intake of *tofu*, *miso* and *natto* intake among postmenopausal women. With intake of *koyadofu* (freeze dried tofu) divided into tertiles, there was no apparent modification of the breast cancer risk among either pre- or postmenopausal women.

We next determined the association between total intake of isoflavone and risk of breast cancer and found a statistically significant inverse association among premenopausal women. Compared with women in the lowest tertile of isoflavone consumption, the top tertile had an adjusted OR of 0.44 (95% CI, 0.22–0.89; trend test *P*=0.02). Among postmenopausal women the similar trend was also observed; however, there was no statistically significant association between consumption of isoflavone and breast cancer risk (Table 5).

## DISCUSSION

The present study found a statistically inverse association between risk of breast cancer and soybean products (*tofu*) or isoflavones intake in Japanese premenopausal women while there was no statistically significant link among their postmenopausal counterparts.

Soybean foods are rich in precursors of the isoflavone daidzein and genistein, which are heterocyclic phenols similar in structure to oestrogens, and it has been hypothesised that a high dietary intake of soybean products might reduce breast cancer risk by interfering with the action of endogenous oestradiol (Messina, 1999). The results are in line with the inverse association between intake of soybean products and breast cancer risk suggested from ecological/cross-sectional studies (Adlercreutz, 1995; Adlercreutz and Mazur, 1997), and also from analytical investigations. Thus, case–control studies have found that soybean food intake was associated with a decreased risk of breast cancer among premenopausal Singapore women (Lee *et al*, 1991), and both pre- and postmenopausal Asian-American women (Wu *et al*, 1996), although a Chinese case–control study failed to detect any protective effects of soybean food (Yuan *et al*, 1995). Cohort studies among Japanese (Hirayama, 1990; Yamamoto *et al*, 2003), Japanese-American (Nomura *et al*, 1978) and Caucasian-American women (Greenstein *et al*, 1996) have also provided some evidence that soybean products may reduce the risk of breast cancer. A prospective study conducted in Japan, however, found no link between soya consumption and breast cancer risk, but in this case the subjects were city residents in Hiroshima or Nagasaki exposed to high doses of ionising radiation and therefore the cohort was unusual (Key *et al*, 1999). A recent cohort study based on public health center in Japan (Yamamoto *et al*, 2003) found frequent *miso soup* and isoflavone consumption to be associated with a reduced risk of breast cancer and the protective effect was stronger in postmenopausal women. However, the FFQ applied included only two items for soybean-ingredient foods (i.e. *miso soup* and soyfoods), making it impossible to investigate differences in effects among types of soybean-ingredient foods.

Isoflavone intake by Japanese is much higher than that by Western populations (Jones *et al*, 1989; Kimira *et al*, 1998). *Tofu*, *miso* and *natto* were main foods containing rich isoflavones. Attributable rate of genistein were *tofu* (49.6%), *miso* (20.9%), *natto* (14.7%) among Japanese. In the present study, *tofu* was protective in premenopausal women, while *atsuage and aburage*, deep fried *tofu* containing much oil, was associated with elevated risk among postmenopausal women. This may be due to fat intake, which can exert an influence on the development of breast cancer among postmenopausal women. Some studies have suggested that high intake of soybean products in premenopausal women may reduce serum oestradiol concentrations, suppress the mid-cycle surge of gonadotropins, and perhaps increase the length of the menstrual cycle (Cassidy *et al*, 1994; Lu *et al*, 1996; Nagata *et al*, 1997).

There is increasing evidence that dietary factors may play a role in the production, metabolism, and bioavailability of sex hormones. Soy-containing diets have long been known to be typical of some ethnic groups who experience low breast cancer risk. Soybeans contain a significant amount of the isoflavones daidzein and genistein, which may exert antioestrogenic effects and protect epithelial tissue from stimulation by endogenous oestrogens. There are several possible mechanisms by which soy isoflavones specifically may modulate the risk of breast carcinoma: (1) increase of serum levels of sex hormone-binding globulin (SHBG) (Adlercreutz *et al*, 1991; Mousavi and Adlercreutz, 1993); (2) downregulation of enzymes involved in oestrogen biosynthesis, such as aromatase (Adlercreutz *et al*, 1993); (3) inhibition of 17*β*-hydroxysteroid dehydrogenase type I (Makela *et al*, 1995); (4) suppression of the gonadotorpins follicule stimulating hormone (FSH) and luteinising hormone (LH); (5) change in intestinal flora, which affect reabsorption of E2 and lower circulating oestrogen levels (Adlercreutz, 1998). Studies examining the effect of a soy protein diet on the menstrual cycle have demonstrated a significant increase in follicular phase length and delay in menstruation, including suppression of midcycle surges of LH and FSH, which potentially may reduce the risk of breast cancer (Cassidy *et al*, 1994; Lu *et al*, 1996; Nagata *et al*, 1998; Kumar *et al*, 2002). Isoflavones have in fact received a great deal of attention due to their antiproliferative properties and these would support the protective effect against breast cancer in premenopausal women observed in the present study. However, clarification of effects in postmenopausal women is still required. In this context it is of interest that isoflavones may exert both oestrogenic and antioestrogenic properties after modification by intestinal bacteria.

Soybeans are a unique dietary source of a group of phytochemicals and several natural anticarcinogens have now been identified in soybeans, such as protease inhibitors, phytates, phytosterols, saponins and lignans. Also, soybeans are an excellent source of dietary fibre and micronutrients, especially calcium. It will be important in future epidemiological studies to investigate the association between intake of soybean products and breast cancer by obtaining a more complete assessment of soybean intake. Further studies are required to confirm the ability of the nutritional profile of soybeans to reduce the risk of breast cancer.

Potential limitations of the present study should be considered. One methodological issue is selection of base population for controls. We applied noncancer patients at ACCH as controls because it is reasonable to assume our cases arise within this population base. Main reasons to visit at ACCH among cases and controls were self/family recommendation, referral from other clinics, and secondary screening after primary screening. Although distributions of these reasons slightly differ, it was considered in statistical analyses. Notable point of our control population is its similarity to general population in terms of exposure of interest, here dietary pattern. We have compared lifestyle characteristics between outpatients in ACCH and the 1231 individuals randomly selected from the general population, and confirmed that they are not substantially different (Inoue *et al*, 1997). Possible bias due to medical background of controls is another potential source of bias; however, our previous report revealed substantially limited impact of it. More than 66% of noncancer outpatients at ACCH did not have any specific medical condition. Remaining 34% of them have specific diseases, but common part of them were benign tumours and/or non-neoplastic polyps (13.1%), mastitis (7.5%), digestive disease (4.1%), or benign gynaecological disease (4.1%) (Hamajima *et al*, 1995). This situation is very different from that in the US, where people visit local general clinics first, and are then referred to hospitals, which function as secondary and/or specific facilities for further medical treatment. We therefore conclude that it is feasible to use noncancer outpatients as controls in epidemiological studies with due consideration of age, sex, season, and reason for visit. The present study was free of recall information bias to the questionnaire because all data were collected prior to diagnoses. Eligible controls were not matched, because our previous study showed that the large number of nonselsected controls gives a steadier estimate than selected, matched controls (Hamajima *et al*, 1994).

Another limitation of the present study included the small number of cases that are more likely to be due to chance leading to a false-positive result. Conversely, small sample may lack sufficient power to detect significant differences. The powers for detection of the OR of 0.5 for higher *vs* lower level of total isoflavone intake were 77% among premenopausal women and 81% among postmenopausal women, respectively (alpha error=0.0500, two-sided). Much larger studies will be required to confirm the impact of soybean products and isoflavone intake on breast cancer risk among Japanese women.

A number of risk factors for breast cancer have been established, most of them related to reproductive events. Evidence from studies of migrant populations, however, has also implicated environmental or lifestyle factors as being of importance and the diet is suspected of playing a role. The present study focused on the Japanese diet, which features a high level of consumption of soybean products and was able to demonstrate that high consumption of soybean products reduces the risk in Japanese premenopausal women. The findings are biologically plausible and suggested a potential beneficial effect of soybean products and isofloavones in the prevention of breast cancer.

## Figures and Tables

**Table 1:** Basic characteristics of cases and controls

	**Cases (*n*=167)**	**Controls (*n*=854)**
*Age (years)*		
30–39	19 (11.4%)	99 (11.6%)
40–49	46 (27.5%)	279 (32.7%)
50–59	54 (32.3%)	280 (32.8%)
⩾60	48 (28.7%)	196 (23.0%)
Mean age (s.d.)	52.7 (10.2)	51.4 (10.5)
		
*Motives for consultation*		
Self-recommendation	47 (28.1%)	286 (33.5%)
Family recommendation	43 (25.8%)	194 (22.7%)
Referral from other clinics	44 (26.4%)	139 (16.3%)
Secondary screening after primary screening	33 (19.8%)	224 (26.2%)
Others	0 (0.0%)	6 (0.7%)
Unknown	0 (0.0%)	5 (0.6%)
		
*Smoking status*		
Never	146 (87.4%)	710 (83.2%)
Ever	9 (5.4%)	51 (6.0%)
Current	12 (7.2%)	91 (10.7%)
Unknown	0 (0.0%)	1 (0.1%)
		
*Drinking status*		
Never	111 (66.5%)	546 (63.9%)
Ever	0 (0.0%)	13 (1.5%)
Current	56 (33.5%)	295 (34.5%)
		
*Exercise*		
No	54 (32.3%)	291 (34.1%)
⩽60 min/week	33 (19.8%)	192 (22.5%)
⩽120 min/week	27 (16.2%)	126 (14.8%)
>120 min/week	47 (28.1%)	225 (26.4%)
Unknown	6 (3.6%)	20 (2.3%)
		
*Mean BMI (s.d.)*	22.9 (3.1)	22.0 (3.0)
Mean of age at first birth (s.d.)	25.8 (3.6)	25.7 (3.5)
Mean of age at menarche (s.d.)	13.5 (1.5)	13.4 (1.6)
		
*Parity*		
Parous	155 (93.4%)	763 (89.7%)
Nulliparous	11 (6.6%)	88 (10.3%)
		
*Menopausal status*		
Premenopausal	79 (47.3%)	414 (48.5%)
Postmenopausal	88 (52.7%)	440 (51.5%)
		
*Age at menopause among postmenopausal women*		
⩽47	24 (27.3%)	105 (23.9%)
48–52	42 (47.7%)	225 (51.1%)
⩾53	22 (25.0%)	83 (18.9%)
Unknown	0 (0.0%)	27 (6.1%)
		
*Family history* a		
No	153 (91.6%)	791 (92.6%)
Yes	14 (8.4%)	63 (7.4%)

s.d.=standard deviation.

a Family history in mother or sisters.

**Table 2:** Distribution of selected dietary variables (intake/day) among breast cancer cases and controls

	**Cases**				**Controls**			
		**Percentile**				**Percentile**		
**Nutritional factors and food items**	**Mean**	**25**	**50**	**75**	**Mean**	**25**	**50**	**75**
*Nutritional factors*								
Energy (kcal)	1831	1568	1791	2038	1859	1593	1835	2093
Total protein (g)	72.1	59.0	70.1	83.0	73.7	60.8	72.1	83.7
Total fat (g)	57.5	43.5	55.0	70.6	59.1	45.5	57.9	70.4
% of energy (%)	27.9	25.0	28.7	31.4	28.4	24.3	28.5	32.3
Carbohydrate (g)	252.9	220.8	243.6	279.7	254.7	217.5	249.8	289.7
								
Total dietary fibre (g)	14.4	11.4	13.6	16.7	14.8	11.5	14.2	17.2
Vitamin C (mg)	161.5	115.6	152.1	194.4	164.9	108.4	146.3	199.3
Vitamin E (mg)	10.1	7.9	9.7	12.1	10.3	8.1	10.0	12.1
Isoflavones (mg)	24.8	15.3	21.3	30.3	27.1	17.0	24.2	32.5
								
*Food items*								
Meat and meat products (g)	60.7	36.4	55.7	77.9	67.0	38.6	61.6	90.0
Fish and fish products (g)	56.5	38.9	52.9	68.6	56.8	36.0	52.9	71.4
Green-yellow vegetables (g)	136.5	78.2	118.9	173.2	144.6	78.2	118.0	180.4
Other vegetables (g)	130.7	82.5	115.7	154.3	135.8	88.2	125.4	172.1
Fruit (g)	164.5	83.4	142.1	217.0	167.1	84.1	143.3	219.7
Dairy products (g)	200.2	87.1	189.3	274.3	199.0	96.8	188.9	265.0
Soybean products (g)	63.4	35.5	53.5	82.4	67.7	39.5	60.5	84.3
Tofu (soybean curd) (g)	39.0	20.0	29.0	50.0	41.7	23.0	35.0	52.0
Miso (fermented soybean paste) (g)	6.3	2.0	5.0	10.0	7.1	5.0	8.0	10.0
Atsuage (deep fried tofu) (g)	7.4	2.5	5.0	10.0	7.3	2.5	5.0	10.0
Aburage (thinly sliced deep fried tofu) (g)	1.7	0.6	1.2	3.0	1.7	0.6	1.2	2.4
Natto (fermented soybeans) (g)	9.2	4.0	7.2	10.0	10.3	4.0	8.0	16.0
Koyadofu (freeze dried tofu) (g)	1.9	0.0	0.0	4.0	2.0	0.0	0.0	4.0

**Table 3:** Odds ratios (ORs) and 95% confidence intervals (CIs) for breast cancer according to food group intake by menopausal status

	**Premenopausal women**				**Postmenopausal women**			
	**OR for tertiles of food group intake (g/1000 kcal)**				**OR for tertiles of food group intake (g/1000 kcal)**			
	**1**	**2**	**3**	***P*-value test**	**1**	**2**	**3**	***P*-value test**
*Meat and meat products*								
Tertile median	24.3	38.4	54.4		13.7	29.4	45.7	
Cases/controls	29/138	31/137	19/139		31/146	39/147	18/147	
OR^a^	1.00	1.02	0.54	0.11	1.00	1.11	0.64	0.24
(95% CI)		(0.54–1.92)	(0.26–1.12)			(0.62–2.02)	(0.32–1.28)	
								
*Fish and fish products*								
Tertile median	14.8	25.6	38.5		18.2	30.6	45.4	
Cases/controls	21/138	29/138	29/138		26/145	37/147	25/148	
OR^a^	1.00	1.53	1.36	0.46	1.00	1.42	0.77	0.42
(95% CI)		(0.74–3.18)	(0.65–2.88)			(0.75–2.66)	(0.39–1.52)	
								
*Vegetables* b								
Tertile median	80.4	125.3	189.6		98.6	145.2	220.5	
cases/controls	22/138	39/138	18/138		31/146	30/146	27/148	
OR^a^	1.00	1.45	0.70	0.38	1.00	1.01	0.85	0.63
(95% CI)		(0.76–2.80)	(0.32–1.50)			(0.54–1.89)	(0.44–1.63)	
								
*Fruit*								
Tertile median	27.5	62.3	115.4		46.3	93.7	158.5	
Cases/controls	28/139	29/136	22/139		24/146	32/147	32/147	
OR^a^	1.00	1.08	0.65	0.24	1.00	1.21	1.38	0.34
(95% CI)		(0.55–2.10)	(0.32–1.33)			(0.62–2.35)	(0.71–2.69)	
								
*Dairy products*								
Tertile median	34.0	98.5	158.5		32.3	103.6	165.5	
Cases/controls	30/139	22/136	27/139		22/146	31/147	35/147	
OR^a^	1.00	0.91	0.73	0.37	1.00	1.54	1.64	0.15
(95% CI)		(0.46–1.79)	(0.37–1.45)			(0.80–2.96)	(0.84–3.20)	
								
*Soybean products*								
Tertile median	17.2	29.7	47.9		20.1	35.3	56.5	
Cases/controls	36/139	23/137	20/138		31/146	28/147	29/147	
OR^a^	1.00	0.60	0.53	0.06	1.00	0.87	0.70	0.28
(95% CI)		(0.30–1.18)	(0.27–1.04)			(0.47–1.61)	(0.37–1.33)	

a Adjusted for age, motives for consultation, smoking, drinking, exercise, energy, family history, age at menarche, parity, age at first full-term pregnancy, BMI and age at menopause for postmenopausal women.

b Included both green-yellow vegetabeles and other vegetables.

**Table 4:** Odds ratios (ORs) and 95% confidence intervals (CIs) for breast cancer according to soybean product intake by menopausal status

	**Premenopausal women**				**Postmenopausal women**			
	**OR for tertiles of soybean product intake (g/1000 kcal)**				**OR for tertiles of soybean product intake (g/1000 kcal)**			
	**1**	**2**	**3**	***P*-value test**	**1**	**2**	**3**	***P*-value test**
*Tofu (soybean curd)*								
Tertile median	8.9	17.1	31.3		10.8	19.6	35.9	
Cases/controls	38/137	20/139	21/137		26/145	36/148	26/147	
OR^a^	1.00	0.44	0.49	0.03	1.00	1.34	0.71	0.34
(95% CI)		(0.22–0.90)	(0.25–0.95)			(0.73–2.44)	(0.36–1.39)	
								
*Miso (fermented soybean paste)*								
Tertile median	1.4	3.4	5.5		1.5	3.9	5.8	
Cases/controls	30/137	30/137	19/136		37/145	23/147	28/147	
OR^a^	1.00	1.14	0.58	0.15	1.00	0.52	0.64	0.11
(95% CI)		(0.59–2.19)	(0.28–1.20)			(0.27–0.98)	(0.34–1.17)	
								
*Atsuage (deep fried tofu)*								
Tertile median	0.0	2.9	5.9		0.0	3.1	6.4	
Cases/controls	29/139	24/137	26/138		20/146	33/146	35/148	
OR^a^	1.00	0.67	0.70	0.31	1.00	1.95	2.28	0.02
(95% CI)		(0.34–1.34)	(0.35–1.38)			(0.98–3.86)	(1.15–4.51)	
								
*Aburage (thinly sliced deep fried tofu)*								
Tertile median	0.0	0.5	1.4		0.3	0.7	1.9	
Cases/controls	23/136	34/136	22/137		22/145	31/147	35/147	
OR^a^	1.00	1.67	1.07	0.97	1.00	1.75	1.62	0.17
(95% CI)		(0.82–3.40)	(0.51–2.26)			(0.89–3.43)	(0.83–3.14)	
								
*Natto (fermented soybeans)*								
Tertile median	1.2	3.7	8.7		1.3	4.0	10.8	
Cases/controls	29/138	26/137	24/137		30/145	30/145	27/148	
OR^a^	1.00	0.89	0.84	0.56	1.00	1.00	0.79	0.47
(95% CI)		(0.46–1.74)	(0.43–1.64)			(0.54–1.87)	(0.41–1.51)	
								
*Koyadofu (freeze dried tofu)*								
Tertile median	0.0	1.9	3.6		0.0	2.1	4.2	
Cases/controls	55/297	6/57	18/60		58/284	14/77	16/79	
OR^a^	1.00	0.68	1.38	0.52	1.00	0.81	0.99	0.84
(95% CI)		(0.27–1.73)	(0.69–2.79)			(0.40–1.64)	(0.50–1.97)	

a Adjusted for age, motives for consultation, smoking, drinking, exercise, energy, family history, age at menarche, parity, age at first full-term pregnancy, BMI and age at menopause for postmenopausal women.

**Table 5:** Odds ratios (ORs) and 95% confidence intervals (CIs) for breast cancer according to isoflavone intake by menopausal status

	**Premenopausal women**				**Postmenopausal women**			
	**OR for tertiles of isoflavone intake (mg/1000 kcal)**				**OR for tertiles of isoflavone intake (mg/1000 kcal)**			
	**1**	**2**	**3**	***P*-value test**	**1**	**2**	**3**	***P*-value test**
Tertile median	7.61	11.87	18.47		8.69	13.59	22.26	
Cases/controls	36/138	24/139	19/137		33/145	29/144	26/151	
OR^a^	1.00	0.62	0.44	0.02	1.00	0.76	0.58	0.09
(95% CI)		(0.32–1.20)	(0.22–0.89)			(0.41–1.40)	(0.30–1.10)	

a Adjusted for age, motives for consultation, smoking, drinking, exercise, energy, family history, age at menarche, parity, age at first full-term pregnancy, BMI and age at menopause for postmenopausal women.
